# The Long-Term Survival of a Patient With Stage IV Renal Cell Carcinoma Following an Integrative Treatment Approach Including the Intravenous α-Lipoic Acid/Low-Dose Naltrexone Protocol

**DOI:** 10.1177/1534735417747984

**Published:** 2017-12-19

**Authors:** Burton M. Berkson, Francisco Calvo Riera

**Affiliations:** 1 Oklahoma State University, Stillwater, OK, USA; 2 The Integrative Medical Center of New Mexico, Las Cruces, NM, USA; 3 Clinic for Autoimmune and Chronic Diseases, León, Spain

**Keywords:** stage IV renal cell carcinoma, metastases to lung, α-lipoic acid, low-dose naltrexone, metabolic control of cancer, hydroxycitrate, vitamin C, integrative medicine

## Abstract

In this case report, we describe the treatment of a 64-year-old male patient diagnosed with metastatic renal cell carcinoma (RCC) in June of 2008. In spite of a left nephrectomy and the standard oncological protocols, the patient developed a solitary left lung metastasis that continued to grow. He was informed that given his diagnosis and poor response to conventional therapy, any further treatment would, at best, be palliative. The patient arrived at the Integrative Medical Center of New Mexico in August of 2010. He was in very poor health, weak, and cachectic. An integrative program—developed by one of the authors using intravenous (IV) α-lipoic acid, IV vitamin C, low-dose naltrexone, and hydroxycitrate, and a healthy life style program—was initiated. From August 2010 to August 2015, the patient’s RCC with left lung metastasis was followed closely using computed tomography and positron emission tomography/computed tomography imaging. His most recent positron emission tomography scan demonstrated no residual increased glucose uptake in his left lung. After only a few treatments of IV α-lipoic acid and IV vitamin C, his symptoms began to improve, and the patient regained his baseline weight. His energy and outlook improved, and he returned to work. The patient had stable disease with disappearance of the signs and symptoms of stage IV RCC, a full 9 years following diagnosis, with a gentle integrative program, which is essentially free of side effects. As of November 2017 the patient feels well and is working at his full-time job.

## Case History

Kidney cancer is among the 10 most common cancers in both men and women. The lifetime risk for developing kidney cancer, which is higher in men than in women, is about 1 in 63 (1.6%), a rate that has been rising since the 1990s.^1^ According to the American Cancer Society, the 5-year survival rate for stage IV renal cancer is just 8%.^1^

**Figure 1.**
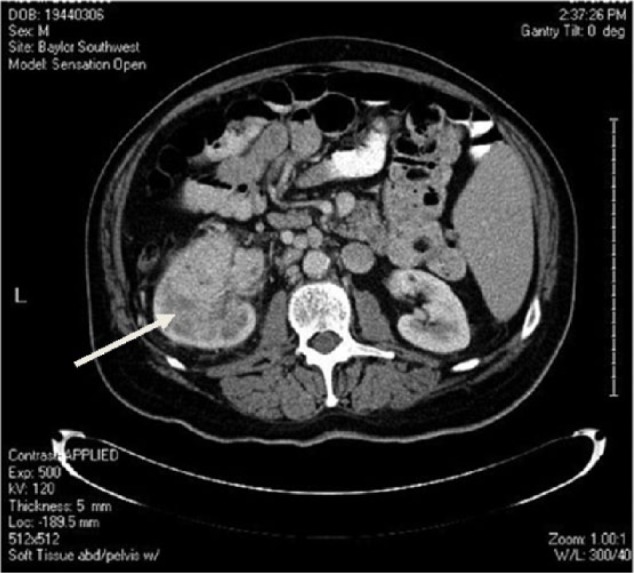
June 2008, CT scan showing a large hyperdense mass (arrow) occupying the mid to lower pole of the left kidney.

**Figure 2.**
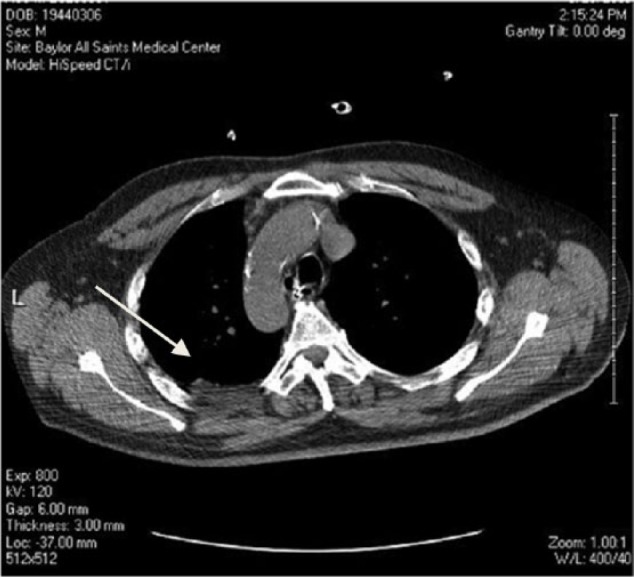
June 2008, CT scan showing a 1-cm noncalcified nodule within the upper lobe of the left lung (see arrow).

The patient subject of this report is a 64-year-old man with a history of fatigue, atherosclerotic vascular disease, prostatitis, joint pains, and myalgias. He was diagnosed with renal cell carcinoma (RCC) with metastases to the lung in early June 2008 after he started to feel vague flank discomfort followed soon afterwards by gross hematuria. He immediately presented to the local emergency room. A computed tomography (CT) scan was performed, which revealed a large hyperdense mass occupying the mid to lower pole of the left kidney (Figure 1) and a 1-cm noncalcified nodule within the upper lobe of the left lung (Figure 2).

After a left nephrectomy, his hematuria resolved, and the patient was started on bevacizumab by his local oncologist for 4 months with no positive results. He was then prescribed sunitinib and sorafenib at a large Texas University cancer center.

Unfortunately, the patient’s condition continued to worsen. He became anemic, leukopenic, and thrombocytopenic and was unresponsive to the antiangiogenic agents. The size of the solitary lung metastasis increased from 1 cm to 8-9 cm on a CT scan.

He was advised that he had exhausted his therapeutic options and that he should consider palliative hospice care given his poor prognosis and lack of response to conventional care.

The patient decided to seek another opinion and traveled to the Integrative Medical Center of New Mexico (IMCNM) in August 2010 where he was seen in consult by one of the authors (BB). At the time of presentation, his review of systems was positive for shortness of breath, seasonal allergic symptoms, heartburn, tinnitus, a decrease in force of urinary stream, insomnia, severe weight loss, flank pain, profound emotional stress, and anxiety. He appeared very thin and frail and weighed 176 pounds, having lost about 30 pounds.

**Figure 3.**
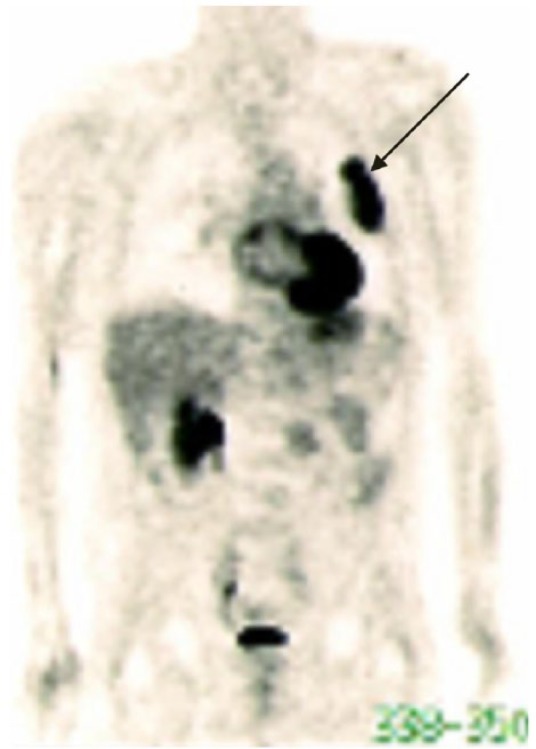
August 2010, a PET/CT scan showed a large pleural based mass in his left upper lung (see arrow).

A full medical workup was conducted including a positron emission tomography (PET)/CT scan that showed a large pleural based mass in his left upper lung (Figure 3).

Since the patient had very few treatment options beyond clinical trials, an integrative medical program was developed and prescribed for him. The purpose of the program was nutritional support, comfort, immune stimulation, and metabolic alteration of the malignant process. The hope was that his disease progression could be slowed and that his life could be prolonged. It was strongly recommended that the patient continue with a board-certified oncologist but he refused because he had been told by his oncologist that he failed conventional treatment regimens.

The key therapeutic agents initially prescribed by BB were intravenous (IV) vitamin C 25 to 50 g every morning and IV racemic α-lipoic acid (ALA) 300 to 600 mg every afternoon after a meal (to prevent hypoglycemia). These therapies were administered at the clinic on an outpatient basis. The oral protocol included low-dose naltrexone (LDN) 4.5 mg at bedtime, the oral Triple Antioxidant Therapy protocol^2,3^ with (1) racemic ALA 300 mg twice daily, (2) selenomethionine 200 µg twice daily, and (3) silymarin 900 mg twice a day along with 3 professional-strength B-50 complex capsules a day. Oral hydroxycitrate (HCA) 500 mg 3 times daily was added to the protocol in September 2013, based on the work of Dr Laurent Schwartz et al.^4,5^

It was also suggested that the patient followed the IMCNM lifestyle program including a strict diet with 4 servings of fresh vegetables a day, very low simple carbohydrate intake, and no processed food, especially preserved animal products. Some organic animal protein was allowed. In addition, an exercise and a stress reducing program were prescribed.

This program had been used frequently at the IMCNM with many patients and was previously reported in the scientific literature in 4 cases of pancreatic cancer and one case of B cell lymphoma.^3,6,7^

After 1week of receiving IV ALA and IV vitamin C, he began to subjectively look and feel better. He reported “increased energy and a new sense of well-being.”

After this 1 week of initial treatment in the author’s clinic, the patient went home and adhered to the programmed lifestyle and supplements, and a local integrative doctor in Fort Worth, Texas, continued the IV ALA infusions twice a week and IV vitamin C twice a week.

**Figure 4.**
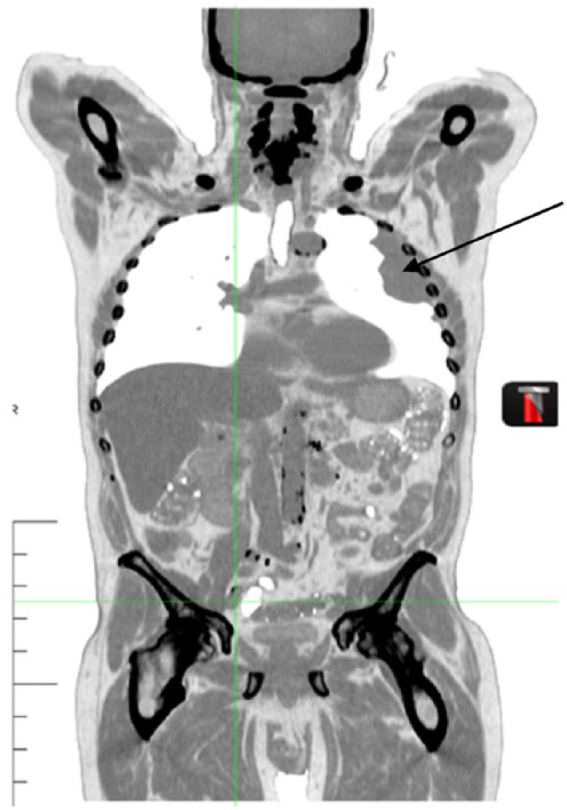
January 2011, a repeat CT scan was performed, and again the mass in the left lung was demonstrated, with no apparent change in size.

The patient continued to visit the IMCNM every 3 months for a week or two of intensive daily IV vitamin C and IV ALA therapy. In January of 2011, he stated that he was beginning to feel healthy again. At that time, he weighed 184 pounds, an 8-pound increase since beginning treatment. The patient continued to go to work throughout the course of his treatment. In January 2011, a repeat PET/CT scan was performed (Figure 4). Again, the mass in the left lung was demonstrated, with no apparent change in size.

In June 2011, another PET/CT scan showed that the upper lung mass was much smaller in size (7.2-4.4 cm; figure not shown).

**Figure 5.**
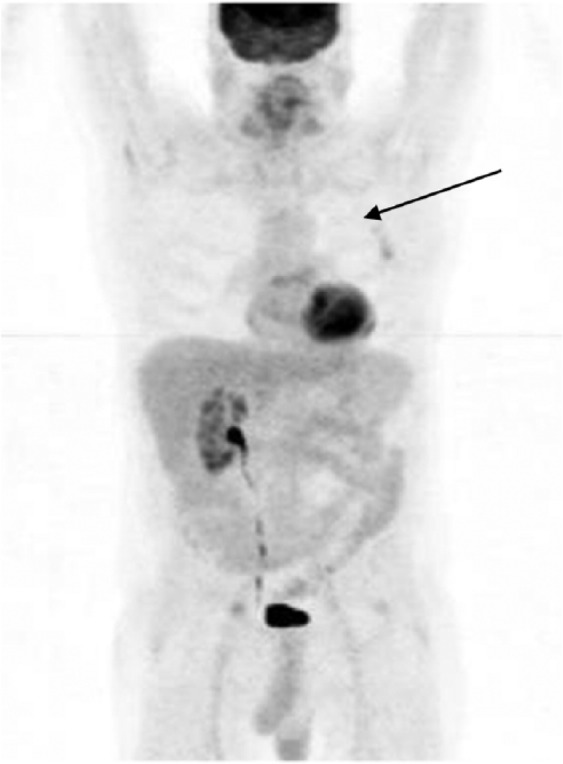
A repeat PET/CT scan in March 2012 showed complete resolution of the upper lung metastasis.

A repeat PET/CT scan in March 2012 showed complete resolution of the upper lung mass (Figure 5).

**Figure 6.**
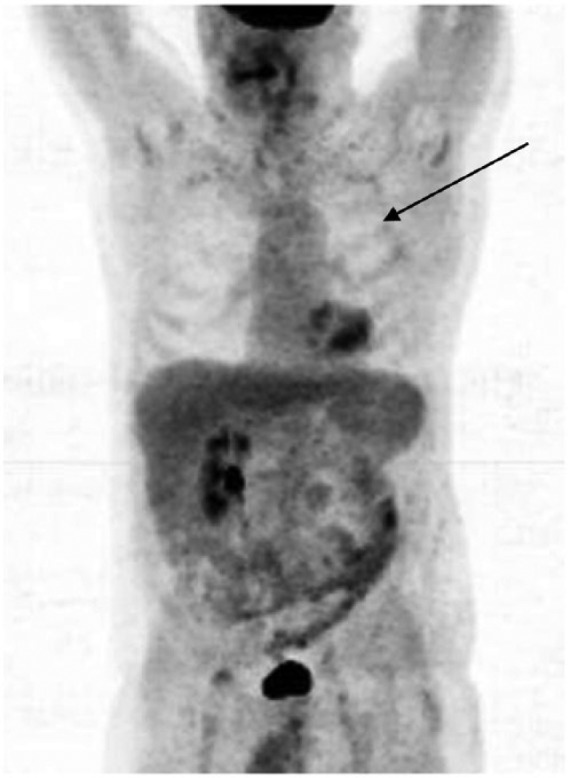
PET/CT scan, September 2013.

**Figure 7.**
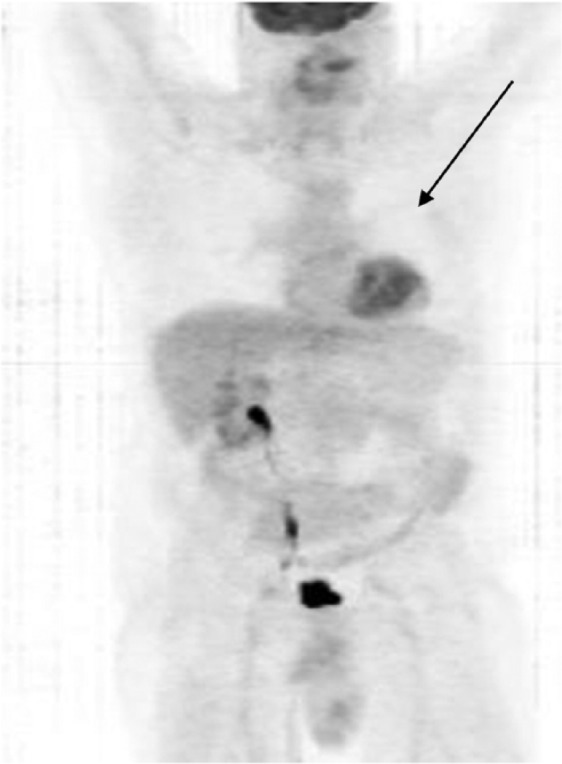
PET/CT scan, January 2014.

**Figure 8.**
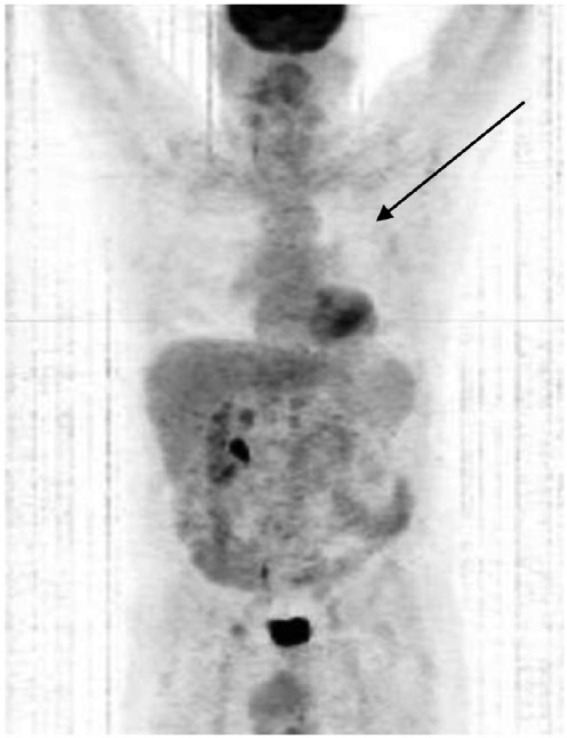
PET/CT scan, August 2014.

Subsequent PET/CT scans in September 2013, January 2014, and August 2014 continued to demonstrate absence of the left lung mass (Figures 6, 7, and 8, respectively). The right kidney contrast in all PET/CT scans was considered by the radiologist to be normal physiologic contrast elimination (left nephrectomy was performed in 2008).

**Figure 9.**
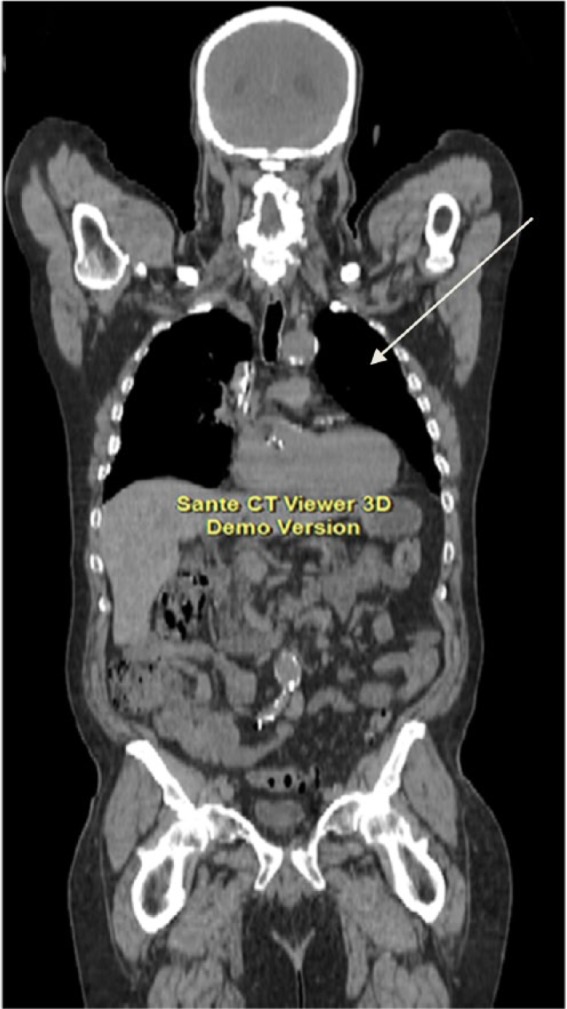
June 2015, CT scan, showing absence of the left pulmonary metastatic mass.

A CT scan of June 2015 (Figure 9) shows no mass in the left lung. As of September 2017, the patient continues on his integrative protocol without changes to his regimen and is alive and in good health. He now weighs 206 pounds, 30 pounds more than when he presented to our clinic.

## Discussion

According to the American Cancer Society, the 5-year survival rate for stage IV RCC is only 8%.^1^

Since the patient had very few treatment options beyond clinical trials, he chose to follow an integrative medical program that included: IV racemic ALA, IV vitamin C, oral LDN, and the oral Triple Antioxidant Therapy protocol^2,7,8^ with oral lipoic acid, selenomethionine, silymarin, and B complex capsules. The patient was also prescribed the IMCNM lifestyle program including a strict diet, exercise, and a stress-reduction program.

This program had been used frequently at IMCNM and was previously reported in the scientific literature in 4 cases of pancreatic cancer and 1 case of B cell lymphoma.^3,6,7^ HCA was added to the protocol in September 2013 based on the work of Dr Laurent Schwartz et al.^4,5^

The first key component in the patient’s treatment protocol was lipoic acid (ALA). IV ALA can reach much higher plasma levels than the oral form, with the oral capsules maintaining levels in between IV infusions. ALA has multiple activities; for instance, it is a powerful antioxidant and heavy metal chelator.^9^ It appears that 3 of its actions are relevant in this case: its anti-inflammatory activity, the effect on mitochondrial metabolism, and its epigenetic activity.

First, ALA may discourage the growth of cancer cells by its action involving the pro-inflammatory transcription factor, nuclear factor κ-light-chain-enhancer of activated B cells (NF-κB).^10^

Unmitigated NF-κB activation can produce proliferation, angiogenesis, mutagenesis, metastasis, and chemo-radio resistance in malignant cells and leave them resistant to apoptosis.^11^ Patients with advanced cancer exhibit greatly elevated markers of oxidative stress and an unrelenting inflammatory process in part due to NF-κB activation.^11^ ALA inhibits NF-κB, blunting these deleterious effects and discouraging the unbridled growth of cancer cells.

Another significant interaction of ALA is with the pyruvate dehydrogenase enzyme complex (PDHC) and its regulatory enzyme pyruvate dehydrogenase kinase (PDK). PDHC consists of 3 mitochondrial enzymes that sit in the intersection of cytoplasm and mitochondria, glycolysis and the Krebs cycle, and anaerobic and aerobic energy metabolism. PDHC converts cytoplasm-generated pyruvate into acetyl CoA, which then enters the Krebs cycle. ALA is the necessary cofactor for PDHC, and without ALA there is no energy produced in the mitochondria.

PDK phosphorylates and inhibits PDHC, regulating its activity. ALA inhibits PDK, and by doing so it further increases PDHC activity.^12,13^

These enzymes are involved in a metabolic peculiarity of cancer cells, the so-called Warburg effect, also called “aerobic glycolysis,” a phenotype rather common to malignancies: cancer cells preferentially metabolize glucose and pyruvate into lactic acid, even in the presence of oxygen.^14^ This increase in glycolysis might favor the formation of amino acid and nucleotide precursors, important for a rapidly proliferating cell, whose importance might offset the disadvantage of a reduced ATP production.^15,16^

McFate et al^17^ have shown that inhibition of PDHC activity contributes to the Warburg metabolic and malignant phenotype in human head and neck squamous cell carcinoma. This inhibition occurred by an enhanced expression of pyruvate dehydrogenase kinase (PDHK). Knockdown of PDHK restored pyruvate dehydrogenase (PDH) activity, reverted the Warburg metabolic phenotype, decreased invasiveness, and inhibited xenograft tumor growth in nude mice.

Clear cell RCC is characterized by the constitutive upregulation of the hypoxia inducible factor-1.^18^ Hypoxia inducible factor-1 has been shown to promote the Warburg effect in several cancers, including clear cell RCC,^18^ in part due to the activation of PDHK (one of its target genes^19^), and subsequent inhibition of PDH. Recently, Lim et al^20^ demonstrated that in this type of cancer, most of the 10 tumor samples studied had an elevated PDHK enzyme level, and a decreased PDHC activity, when compared with patient-matched normal tissue.

Schell et al^21^ furthered this idea by implying that the inhibition of the Warburg effect in colon cancer cells was associated with decreased cancer cell xenograft growth in nude mice.

Studying a related issue concerning these enzymes, Kaplon et al^22^ demonstrated that PDH is a crucial mediator of malignant cell senescence induced by BRAF^V600E^, a protein kinase and oncogene that is often mutated in melanoma and other cancers. This BRAF^V600E^-induced senescence was accompanied by simultaneous inhibition of PDHK. Enforced normalization of PDHK inhibited PDH and abolished oncogene-induced senescence, thereby allowing BRAF^V600E^-driven melanoma growth.

Since ALA inhibits PDHK and activates PDHC, the metabolic peculiarity of cancer cells described by Warburg may be mitigated, and it is likely that the overall cancer growth program may be altered. It is also possible, according to Kaplon’s work, that tumor cell senescence may also be promoted by ALA’s action on these enzymes.

Another potential antineoplastic action of ALA concerns its epigenetic activity. ALA can inhibit histone deacetylase (HDAC) activity in human tumor cells.^23,24^ Histone acetylation and deacetylation are important components in gene regulation.

An active avenue of cancer research involves inhibitors of HDACs, with drugs such as vorinostat.

Recently, it has been found that PDH, thought to be an exclusive mitochondrial enzyme, is also present and functional in the nucleus, probably translocated from the mitochondrion.^25^ Inhibition of nuclear PDH in isolated nuclei decreased the acetylation of histone lysine residues. This nuclear PDH has also lipoic acid as cofactor, so ALA provides a source for nuclear acetyl-CoA synthesis required for histone acetylation and epigenetic regulation.

These epigenetic modifications give ALA the capacity of influencing, at a genetic level, tumor behavior and growth. In other words, cancer is not only a genetic disease but is also a metabolic disease. ALA seems to address both of these components.

IV vitamin C was also part of the patient’s treatment protocol. Integrative medical doctors have administered this agent for many years with some positive case history reports. Some of these results include reversal of pulmonary metastases from RCC and from hepatocellular carcinoma.^26,27^

HCA is an extract from the citrus fruit, *Garcinia cambogia*. Not only it inhibits pancreatic α-amylase and intestinal α-glucosidase, but also inhibits ATP-citrate lyase,^28^ a cytoplasmic enzyme that catalyzes the generation of acetyl-CoA from mitochondrial-generated citrate.^29^ Acetyl-CoA is a vital building block for the endogenous biosynthesis of fatty acids, cholesterol, and isoprenoids.

Studies with C-14 glucose have shown that in cancer cells most fatty acids come from a high rate of de novo synthesis, necessary for a very active membrane biogenesis,^30^ and inhibition of ATP-citrate lyase produced inhibition of tumor proliferation in vitro and reduced in vivo xenograft tumor growth.^29^

The authors added HCA to their regime in 2013 at the suggestion of Dr Laurent Schwartz, who showed that HCA was synergistic with ALA to reduce tumor growth in vitro and in vivo.^4,5^

ATP-citrate lyase increases histone acetylation in response to growth factor stimulation and during differentiation.^31^ As is the case with ALA,^23-25^ HCA also has an action on cellular metabolism and promotes epigenetic changes. These epigenetic changes could be relevant to the action of these 2 molecules, and it is a fertile ground for further future research.

LDN was yet another key ingredient in this treatment program. Naltrexone is an opioid antagonist that was originally FDA (Food and Drug Administration) approved in 1984 for heroin addiction.

Zagon and McLaughlin^32,33^ documented the presence of the opioid growth factor receptor (OGFr) axis in a number of human cancers including neuroblastoma, pancreatic, colon, breast, renal, squamous cell carcinoma of the head and neck, and hepatocellular adenoma. In vitro and in vivo studies revealed that opioid growth factor (OGF; also called met-enkephalin), while requiring the presence of OGFr, inhibited cell proliferation in culture and when transplanted into nude mice.^32,33^

They also demonstrated that opioid antagonists, like naltrexone, block the endogenous ligand-receptor interaction. Studies using naltrexone to block the receptor showed that high-dose naltrexone blocked the OGFr for a longer duration than LDN did. The duration of time that the receptor was blocked yielded 2 totally different results. Low dosages of the opioid antagonist administered once daily blocked the receptor for short bursts of time and resulted in suppressed cancer cell proliferation and decreased growth of malignant cells in culture and of tumors in mice. These cancer-bearing mice lived longer than controls. Conversely, higher dosages of naltrexone that blocked the receptor for an entire 24-hour period resulted in enhanced growth of the malignant cells and decreased survival of cancer-afflicted mice. This apparent contradiction was solved when it was determined that short-term opioid receptor blockade paradoxically upregulated enkephalin peptide production. The increased levels of circulating enkephalins subsequently bound to OGFr and inhibited cancer cell proliferation.

Administration of low dosages of naltrexone several times daily in order to continuously block the opioid receptor resulted in increased growth of malignant cells. This confirmed that it was the duration of the OGFr blockade, and not the dosage of the naltrexone, that blocked accelerated cancer cell growth.^32,33^

In humans, nocturnally administered LDN blocks endogenous opiate receptors for a short period of only a few hours.^34^ Due to this receptor blockade, the body perceives a deficiency of endogenous opioids. As a result, endogenous opioid production is dramatically increased (including the production of OGF), OGFr production is upregulated, and the affinity between OGF and OGFr is increased. These endogenous opiates act on 2 sites: on the tumor cells directly, inhibiting their growth, and on the immune system, upregulating it.^34^

Dr Bernard Bihari first used LDN to treat patients with AIDS in the 1980s after discovering the preclinical research of Zagon et al. Given his promising results and based on Zagon’s work, he later used LDN in the treatment of cancer patients.^35^ At the IMCNM, we have used LDN in most patients with malignancies.^3,6,7^

## Conclusion

In this case report, we describe the treatment of a 64-year-old patient who was diagnosed with metastatic RCC in June of 2008. Following a left nephrectomy and the standard oncological protocols consisting of bevacizumab, sunitinib, and sorafenib, the patient became leukopenic and thrombocytopenic and could not tolerate any further conventional treatment.

Furthermore, with the standard protocols, his cancer progressed and he was informed that given his diagnosis and poor response to conventional therapy, any further treatment would, at best, be palliative.

The patient arrived at the IMCNM in August of 2010 as a last resort. He was weak, cachectic, physically and emotionally exhausted, and was experiencing almost constant shortness of breath, flank and abdominal pain, and nausea. An integrative program developed by one of the authors (BB)^3,6,7^ using IV α-lipoic acid, IV vitamin C, low-dose naltrexone, and HCA, and a healthy life style program was initiated.

Lipoic acid potentially has a central role as it addresses 2 main cancer aspects: metabolic (by its action on the PDHC and the Warburg effect) and epigenetic (by its action on HDAC activity).

From August 2010 to the present (November 2017), the patient’s RCC with metastasis to the left lung has been followed closely using CT and PET/CT imaging. After only a few treatments of IV ALA and IV vitamin C, his symptoms began to improve, and the patient actually regained his original baseline weight, his energy and outlook improved, his dyspnea resolved, and even returned to work. His most recent PET/CT scan demonstrated normal glucose uptake in his left lung (Figure 9). The patient had stable disease with an elimination of the signs and symptoms of stage IV RCC, a full 9 years following diagnosis, with a gentle integrative program, which was essentially free of side effects in his case.

However, from the authors’ experience, when the treatment protocol is halted, in many cases, the cancer growth resumes. Thus, the ALA/N (α-lipoic acid/low-dose naltrexone) protocol appears to induce tumor reduction/dormancy rather than cure the disease process. Further multicenter studies are warranted to assess the success of this treatment regimen and long-term results on a wider population.

## References

[1] American Cancer Society. Home page. www.cancer.org. Accessed December 1, 2017.

[2] Berkson BM A conservative triple antioxidant approach to the treatment of hepatitis C. Combination of alpha lipoic acid (thioctic acid), silymarin, and selenium: three case histories. Med Klin (Munich). 1999; 94(suppl 3):84-89. 10554539 10.1007/BF03042201

[3] Berkson BM Rubin D Berkson AJ The long-term survival of a patient with pancreatic cancer with metastases to the liver after treatment with the intravenous alpha-lipoic acid/low-dose naltrexone protocol. Integr Cancer Ther. 2006;5:83-89. 16484716 10.1177/1534735405285901

[4] Guais A Baronzio G Sanders E et al Adding a combination of hydroxycitrate and lipoic acid (METABLOC™) to chemotherapy improves effectiveness against tumor development: experimental results and case report. Invest New Drugs. 2012;30:200-211. 20931262 10.1007/s10637-010-9552-x

[5] Schwartz L Buhler L Icard P Lincet H Steyaert JM Metabolic treatment of cancer: intermediate results of a prospective case series. Anticancer Res. 2014;34:973-980. 24511042

[6] Berkson BM Rubin D Berkson AJ Reversal of signs and symptoms of a B-cell lymphoma in a patient using only low dose naltrexone. Integr Cancer Ther. 2007;6:293-296. 17761642 10.1177/1534735407306358

[7] Berkson BM Rubin D Berkson AJ Revisiting the ALA/N (alpha-lipoic acid/low-dose naltrexone) protocol for people with metastatic and nonmetastatic pancreatic cancer: a report of 3 new cases. Integr Cancer Ther. 2009;8:416-422. 20042414 10.1177/1534735409352082

[8] Berkson BM Thioctic acid in treatment of hepatotoxic mushroom (Phalloides) poisoning. N Engl J Med. 1979;300:371. 10.1056/NEJM197902153000723 366411

[9] Gorąca A Huk-Kolega H Piechota A Kleniewska P Ciejka E Skibska B Lipoic acid—biological activity and therapeutic potential. Pharmacol Rep. 2011;63:849-858. 22001972 10.1016/s1734-1140(11)70600-4

[10] Ying Z Kampfrath T Sun Q Parthasarathy S Rajagopalan S Evidence that α-lipoic acid inhibits NF-κB activation independent of its antioxidant function. Inflamm Res. 2011;60:219-225. 20927568 10.1007/s00011-010-0256-7 PMC5832356

[11] Grivennikov SI Greten FR Karin M Immunity, inflammation, and cancer. Cell. 2010;140:883-899. 20303878 10.1016/j.cell.2010.01.025 PMC2866629

[12] Korotchkina LG Patel MS Pyruvate dehydrogenase complex regulation and lipoic acid. In: Patel MS Packer L eds. Lipoic Acid. Energy Production, Antioxidant Activity, and Health Effects. Boca Raton, FL: CRC Press; 2008:149-165.

[13] Korotchkina LG Sidhu S Patel MS R-lipoic acid inhibits mammalian pyruvate dehydrogenase kinase. Free Radic Res. 2004;38:1083-1092. 15512796 10.1080/10715760400004168

[14] Warburg O On the origin of cancer cells. Science. 1956;123:309-314. 13298683 10.1126/science.123.3191.309

[15] DeBerardinis RJ Lum JJ Hatzivassiliou G Thompson CB The biology of cancer: metabolic reprogramming fuels cell growth and proliferation. Cell Metab. 2008;7:11-20. 18177721 10.1016/j.cmet.2007.10.002

[16] Heiden MGV Cantley LC Thompson CB Understanding the Warburg effect: the metabolic requirements of cell proliferation. Science. 2009;324:1029-1033. 19460998 10.1126/science.1160809 PMC2849637

[17] McFate T Mohyeldin A Lu H et al Pyruvate dehydrogenase complex activity controls metabolic and malignant phenotype in cancer cells. J Biol Chem. 2008;283:22700-22708. 18541534 10.1074/jbc.M801765200 PMC2504897

[18] Semenza GL HIF-1 mediates the Warburg effect in clear cell renal carcinoma. J Bioenerg Biomembr. 2007;39:231-234. 17551816 10.1007/s10863-007-9081-2

[19] Kim JW Tchernyshyov I Semenza GL Dang CV HIF-1-mediated expression of pyruvate dehydrogenase kinase: a metabolic switch required for cellular adaptation to hypoxia. Cell Metab. 2006;3:177-185. 16517405 10.1016/j.cmet.2006.02.002

[20] Lim HY Yip YM Chiong E et al Metabolic signatures of renal cell carcinoma. Biochem Biophys Res Commun. 2015;460:938-943. 25839656 10.1016/j.bbrc.2015.03.130

[21] Schell JC Olson KA Jiang L et al A role for the mitochondrial pyruvate carrier as a repressor of the Warburg effect and colon cancer cell growth. Mol Cell. 2014;56:400-413. 25458841 10.1016/j.molcel.2014.09.026 PMC4268416

[22] Kaplon J Zheng L Meissl K et al A key role for mitochondrial gatekeeper pyruvate dehydrogenase in oncogene-induced senescence. Nature. 2013;149:109-112. 10.1038/nature12154 23685455

[23] Van de Mark K Chen JS Steliou K Perrine SP Faller DV Alpha-lipoic acid induces p27Kip-dependent cell cycle arrest in non-transformed cell lines and apoptosis in tumor cell lines. J Cell Physiol. 2003;194:325-340. 12548552 10.1002/jcp.10205

[24] Dashwood RH Ho E Dietary histone deacetylase inhibitors: from cells to mice to man. Semin Cancer Biol. 2007;17:363-369. 17555985 10.1016/j.semcancer.2007.04.001 PMC2737738

[25] Sutendra G Kinnaird A Dromparis P et al A nuclear pyruvate dehydrogenase complex is important for the generation of acetyl-CoA and histone acetylation. Cell. 2014;158:84-97. 24995980 10.1016/j.cell.2014.04.046

[26] Sao MS Kim JK Shim JY High-dose vitamin C promotes regression of multiple pulmonary metastases originating from hepatocellular carcinoma. Yonsei Med J. 2015;56:1449-1452. 26256994 10.3349/ymj.2015.56.5.1449 PMC4541681

[27] Fritz H Flower G Weeks L et al Intravenous vitamin C and cancer: a systematic review. Integr Cancer Ther. 2014;13:280-300. 24867961 10.1177/1534735414534463

[28] Zu XY Zhang QH Liu JH et al ATP citrate lyase inhibitors as novel cancer therapeutic agents. Recent Pat Anticancer Drug Discov. 2012;7:154-167. 22339355 10.2174/157489212799972954

[29] Hatzivassiliou G Zhao F Bauer DE et al ATP citrate lyase inhibition can suppress tumor cell growth. Cancer Cell. 2005;8:311-321. 16226706 10.1016/j.ccr.2005.09.008

[30] Ookhtens M Kannan R Lyon I Baker N Liver and adipose tissue contributions to newly formed fatty acids in an ascites tumor. Am J Physiol. 1984;247(1 pt 2):R146-R153. 6742224 10.1152/ajpregu.1984.247.1.R146

[31] Wellen KE Hatzivassiliou G Sachdeva UM Bui TV Cross JR Thompson CB ATP-citrate lyase links cellular metabolism to histone acetylation. Science. 2009;324:1076-1080. 19461003 10.1126/science.1164097 PMC2746744

[32] Zagon IS Donahue R McLaughlin PJ Targeting the opioid growth factor: opioid growth factor receptor axis for treatment of human ovarian cancer. Exp Biol Med (Maywood). 2013;238:579-587. 23856908 10.1177/1535370213488483

[33] Donahue RN McLaughlin PJ Zagon IS Low-dose naltrexone targets the opioid growth factor-opioid growth factor receptor pathway to inhibit cell proliferation: mechanistic evidence from a tissue culture model. Exp Biol Med (Maywood). 2011;236:1036-1050. 21807817 10.1258/ebm.2011.011121

[34] Hytrek SD McLaughlin PJ Lang CM Zagon IS Inhibition of human colon cancer by intermittent opioid receptor blockade with naltrexone. Cancer Lett. 1996;101:159-164. 8620464 10.1016/0304-3835(96)04119-5

[35] Bihari B Bernard Bihari, MD: low-dose naltrexone for normalizing immune system function. Altern Ther Health Med. 2013;19:56-65. 23594453

